# Androgenicity in Young Women and Development of Metabolic Syndrome Before Menopause: The CARDIA and CARDIA Women’s Studies

**DOI:** 10.1210/jendso/bvad174

**Published:** 2023-12-28

**Authors:** Thanh-Huyen T Vu, Amber Pirzada, Cora E Lewis, Pamela J Schreiner, Kiang Liu, Barbara Sternfeld, Ronit Calderon-Margalit, Sharon S Merkin, Melissa Wellons, O Dale Williams, Catherine Kim, David S Siscovick, Martha L Daviglus

**Affiliations:** Department of Preventive Medicine, Northwestern University, Feinberg School of Medicine, Chicago, IL 60611, USA; Institute for Minority Health Research, University of Illinois at Chicago, Chicago, IL 60612, USA; Department of Epidemiology, University of Alabama at Birmingham, Birmingham, AL 35233, USA; Diabetes Research and Training Center School of Medicine, University of Alabama at Birmingham, Birmingham, AL 35233, USA; Division of Epidemiology and Community Health, University of Minnesota, Minneapolis, MN 55454, USA; Department of Preventive Medicine, Northwestern University, Feinberg School of Medicine, Chicago, IL 60611, USA; Division of Research, Kaiser Permanente, Oakland, CA 94612, USA; Hebrew University-Hadassah Braun School of Public Health and Social Medicine, Jerusalem 91120, Israel; Division of Geriatrics, Geffen School of Medicine at UCLA, Los Angeles, CA 90095, USA; Division of Diabetes, Endocrinology, & Metabolism, Vanderbilt Eskind Diabetes Clinic, Vanderbilt University Medical Center, Nashville, TN 37232, USA; Robert Stempel College of Public Health and Social Work, Florida International University, Miami, FL 33174, USA; Medical School and School of Public Health, University of Michigan, Ann Arbor, MI 48109, USA; New York Academy of Medicine, New York, NY 10029, USA; Cardiovascular Health Research Unit, Department of Medicine, University of Washington, Seattle, WA 98195, USA; Department of Epidemiology, University of Washington, Seattle, WA 98195, USA; Department of Preventive Medicine, Northwestern University, Feinberg School of Medicine, Chicago, IL 60611, USA; Institute for Minority Health Research, University of Illinois at Chicago, Chicago, IL 60612, USA

**Keywords:** total testosterone, free testosterone, sex hormone binding globulin, metabolic syndrome, pre-menopause, prospective study

## Abstract

**Context:**

There are no reported data from prospective long-term studies on the relation of androgen levels in young women with development of metabolic syndrome (MetS) before menopause.

**Objective:**

We investigated associations of androgens and SHBG with incident MetS during 23 years of follow-up.

**Methods:**

We included 366 White and 375 Black women ages 20 to 32 years participating in the CARDIA study and CARDIA Women’s study, free of MetS at baseline examination (1987-1988), and premenopausal 23 years later. Androgens and SHBG were categorized into quartiles. MetS was defined according to the American Heart Association/National Heart, Lung, and Blood Institute 2009 Joint Scientific Statement. Cox proportional hazards models were used.

**Results:**

By year 23, 30% of women developed MetS. Adjusting for baseline age, race, and education, hazard ratios (95% CI) of developing MetS were 1.46 (1.02-2.10) and 2.22 (1.53-3.21) for women in the highest vs lowest total testosterone (T) and free T quartile, respectively. The hazards of developing MetS were 47%, 59%, and 53% lower for women with SHBG in the second, third, and fourth quartiles (vs lowest quartile), respectively. Associations were attenuated for total T with further adjustments for smoking, physical activity, menstrual status, oral contraceptive/hormone (OCHM) use, insulin level, oligomenorrhea, and age at menarche, but remained statistically significant for free T and SHBG. Associations were similar for both Blacks and Whites, and OCHM nonusers, but not for OCHM users.

**Conclusion:**

High androgenicity in young premenopausal women is associated with higher risk of future MetS, suggesting that early assessment of androgens may contribute to prevention.

Metabolic syndrome (MetS), a constellation of metabolic risk factors, including atherogenic dyslipidemia, elevated blood pressure, elevated serum glucose, and abdominal adiposity, is strongly associated with greater risk of type 2 diabetes [1, 2], and cardiovascular disease (CVD) morbidity [3-5] and mortality [4-6]. The prevalence of MetS was about 24% of US women during 1988 through 1994 [7] and has increased to nearly 37% in 2015 through 2016 [8].

Previous studies have reported a higher incidence of MetS and its individual components among women with polycystic ovarian syndrome (PCOS), which is characterized by reduced chronic ovulation, hyperandrogenism, and polycystic ovaries [9-12]. A higher risk of cardiometabolic diseases has also been found in women with elevated androgen levels [13-25]. Androgen disorders, including PCOS and congenital adrenal hyperplasia, are among the most common endocrinopathies among women [26, 27]. However, the long-term association of androgen levels—indicated by total testosterone (T), free T, and SHBG—with development of MetS among premenopausal women, especially at young ages from general populations, has not been fully investigated. Previous studies on the association of androgenicity and MetS in premenopausal women were either cross-sectional [17, 21-23], reported the associations in older premenopausal women [20, 24], and/or focused on homogenous populations [18, 24, 25]. Thus, a better understanding of the association of androgens measured early in adulthood and the development of MetS before menopause can have important implications for early identification of women at risk for MetS, the prevention of MetS, and ultimately of diabetes and CVD morbidity and mortality.

We used longitudinal data from the biracial Coronary Artery Risk Development in Young Adults (CARDIA) and CARDIA Women’s Studies to examine the association of total T, free T, and SHBG in young women with decades-long risk of incident MetS before menopause.

## Materials and Methods

### Study Sample

The study sample was drawn from female participants in the CARDIA study, a prospective, multicenter, population-based investigation of the evolution of coronary artery disease risk factors in young adults. Details of the CARDIA study design have been previously reported [28]. Briefly, the cohort includes 5115 men and women (51% of eligible persons contacted) aged 18 to 30 years at baseline (year 0; 1985-1986) who were recruited from 4 metropolitan areas (Birmingham, Alabama; Chicago, Illinois; Minneapolis, Minnesota; and Oakland, California). Participants were reexamined at years 2 (1987-1988), 5 (1990-1991), 7 (1992-1993), 10 (1995-1996), 15 (2000-2001), 20 (2005-2006), and 25 (2010-2011) with follow-up rates of 91%, 86%, 81%, 79%, 74%, 72%, and 72%, respectively, of the surviving cohort members. Following the year 15 examination, a subset of female participants were recruited for a year 16 examination (2002-2003) as part of the CARDIA Women’s Study (CWS), an ancillary study designed to examine cross-sectional, prospective, and longitudinal relations between androgens, polycystic ovaries, and cardiovascular risk and subclinical cardiovascular disease [29]. Both the CARDIA and CWS studies were approved by the institutional review boards at each center and written informed consent was obtained from all participants.

There were 838 premenopausal CARDIA women at the year 25 examination for whom data on androgens and SHBG levels at year 2 were available. Of those, 97 were excluded hierarchically from the current analyses because of the following reasons at year 2: pregnant (n = 31), having prevalent MetS (n = 16), having extreme hormone levels (total T > 200 ng/dL, free T > 2.0 ng/dL) (n = 6), or missing values on covariates (race [n = 1], education [n = 2], physical activity [n = 2], smoking [n = 4], insulin [n = 3], current menstrual status [n = 16], and age of menarche [n = 16]). Thus, the final sample consisted of 741 premenopausal women (375 Blacks and 366 Whites) aged 20 to 32 years and without MetS at the year 2 examination. All women had data on incident MetS at follow-up examinations (years 5, 7, 10, 15, 20, and 25).

### Measurement of Androgens

Hormone measurements were performed for female CARDIA participants using stored serum specimens from years 2, 10, and 16. Specimens were not timed to the menstrual cycle; however, at year 16, 64.6% of the CWS sample were drawn on days 2 through 10 of the menstrual cycle. All serum specimens were batched for each participant assayed.

Three indicators of androgenicity, total T, free T, and SHBG, were measured using stored sera. Total T was measured using a competitive immunoassay (Bayer Diagnostics) that uses direct chemiluminescent technology on the 180 Automated Chemiluminescent System. SHBG was measured using equilibrium dialysis, which estimated the amount of testosterone capable of being bound by SHBG in the sample. Based on the measured total T and SHBG levels, free T was calculated using the method described by Pearlman [30]. Because the minimum level of detectability for the total T assay was 10 ng/dL, all assay values ≤10 were set to 5 ng/dL. Levels of these 3 markers of androgenicity were categorized into quartiles (Q1 to Q4) for this study.

### Definition of Metabolic Syndrome

According to the American Heart Association/National Heart, Lung, and Blood Institute 2009 Joint Scientific Statement [5], MetS among women is defined as the presence of 3 or more of the following criteria: triglycerides ≥150 mg/dL, high-density lipoprotein (HDL) cholesterol <50 mg/dL, blood pressure (systolic/diastolic) ≥130/85 mm Hg or on blood pressure medication, fasting glucose ≥100 mg/dL or on medication for diabetes, and waist circumference ≥88 cm. Incident MetS at examination years 5, 7, 10, 15, 20, and 25 was the main outcome of this study.

### Measurement of MetS Components

Data on the 5 components of MetS were collected at years 2, 5, 7, 10, 15, 20, and 25 follow-up clinical examinations. Participants were asked to fast for 12 hours and to avoid smoking and heavy physical activity for 2 hours before each examination. Three seated blood pressure measurements were obtained with either a random-zero sphygmomanometer (baseline to year 15) or Omron sphygmomanometer (years 20 and 25), and the average of the second and third readings was used. Waist circumference was measured in duplicate at the minimum abdominal girth (midway between the iliac crest and the xiphoid process regardless of physical minimum). Serum, plasma, and buffy coats were isolated from venous blood samples and stored at −70 °C for analyses. Serum glucose was measured by the hexokinase method. Plasma total and HDL cholesterol and triglycerides were determined by enzymatic procedures using standard laboratory methods [31]. Because glucose levels were not measured at year 2, glucose levels obtained at year 0 were used in place of year 2 to determine prevalent MetS at baseline for this study.

### Covariates

The covariates examined in this study were those known or hypothesized to influence either the androgen levels or the development of MetS. Specifically, information on age, race (Black and White), educational attainment (years of education), smoking status (current smokers compared with former/never smokers), physical activity score calculated as the sum of moderate and heavy exercise in exercise units [32], age at menarche, current menstrual status (currently menstruating or not), and oral contraceptive/exogenous hormone (OCHM) use were obtained by self-report. Body weight was measured with participants wearing light clothing; body height was measured without shoes. Body mass index (BMI) was then computed as weight divided by height squared (kg/m^2^). Because serum insulin levels were not collected at year 2, data for year 0 were used as a covariate in this study.

At the year 16 examination, participants were also asked to report clinical symptoms including oligomenorrhea and hirsutism (ie, clinical evidence of hyperandrogenism) in the past (ages 20-30 years) and current (ages 34-46 years). Those who indicated either regular or irregular menstrual cycles ≥34 days were considered to have oligomenorrhea, and those reporting unwanted hair growth (excluding the lower leg and underarm) were considered to have hirsutism. Those with oligomenorrhea and hyperandrogenism (ie, either biochemical evidence of hyperandrogenism or hirsutism) were considered to have PCOS.

### Data Analyses

Sociodemographic and selected characteristics were compared across quartiles of each androgen levels. Chi-square (for categorical variables) or F-tests (for continuous variables) were used to assess statistical significance of differences across quartiles.

Cox proportional hazards regression analysis was used to calculate hazard ratios for the development of MetS by androgens and SHBG levels measured at year 2. Because the first available hormone measurements were from the CARDIA year 2 examination, we considered year 2 as baseline. Women who did not develop MetS during 23 years of follow-up were right-censored at end of follow-up. Two models were used for each marker of androgenicity. Model I included adjustment for baseline (year 2) age, race (Black vs White), and years of education. Model II included all variables in model I with further adjustment for baseline smoking status, physical activity, current menstrual status, OCHM use, oligomenorrhea symptom, insulin level, and age at menarche to examine the potential impact of these variables on the androgenicity-MetS relationship. Hazard ratios for incident MetS were compared by quartiles of androgens/SHBG using the first (lowest) quartile as the reference group. Linear trends were tested using the significant level for coefficients across quartiles of androgens in Cox regression models. Additionally, we reran model II, including BMI, to examine whether a higher BMI could explain the relationship between androgenicity and MetS (model III). Because diabetes medication use may affect the onset of MetS [33], we also reran models excluding participants who reported medication use in any of the 8 examinations to mitigate any potential impact of diabetes medication use on the associations.

Similar Cox models were used for analysis of Blacks and Whites separately to examine potential differences in the association of androgenicity and MetS by race. Furthermore, because the use of OCHM may affect androgens/SHBG levels [34, 35], analyses were repeated separately for OCHM users and nonusers. Interaction terms of androgens/SHBG with race and with OCHM use were also examined. In addition, to further explore the relationship between androgens/SHBG levels and MetS, associations of androgens/SHBG levels with individual components of MetS were analyzed. Tests for the proportional hazards’ violations for quartiles of androgens/SHBG were not statistically significant.

### Sensitivity Analyses

To examine how changes in covariates over time (such as education, smoking, physical activity, and OCHM use, obtained at examination years 2, 5, 7, 10, 15, and 20) confound the association of androgens/SHBG levels and MetS, Cox models with time-varying covariates were performed in 528 women (after further exclusions of missing covariates in subsequent examinations). Weight change between examinations was also included in the models as a time-varying covariate. Analyses using androgens/SHBG measured at examination year 10 and especially at examination year 16 (where specimens were drawn on days 2-10 of the menstrual cycle if possible) were also conducted to examine whether patterns of associations with incident MetS (if any) were similar to those based on examination year 2 measures, even though the follow-up was shorter. Finally, supplemental analyses were performed using information on PCOS from the year 2 and year 16 examinations for women without PCOS, to examine whether the relationships between levels of androgens and MetS is independent of the presence of PCOS.

All analyses were conducted in SAS 9.4 with SAS/STAT 15.2 (SAS Institute Inc., Cary, NC).

## Results

At baseline for this analysis, study participants had a mean age of 26 years with an average of 14.3 years of education. About 51% of women were Black. In general, mean educational attainment was lower with higher free T levels and the proportion of women who were currently using any oral contraceptive or exogenous hormones (only 4 women in this analysis sample used exogenous hormones) was lower with higher total T and free T levels, whereas mean BMI and the proportion who were current smokers were higher (Table 1). For example, from lowest to highest total T quartiles, 11.7%, 17.0%, 24.1%, and 36.9% of women, respectively, were current smokers at baseline. Conversely, educational attainment and oral contraceptive use were higher and mean BMI, insulin levels, and current smoker percentages were lower with higher SHBG levels. The prevalence of baseline PCOS was highest in the fourth quartile of total T and free T, and in the lowest quartile of SHBG. Insulin resistance was also highest in the highest free T quartile and lowest SHBG quartile. No significant differences across quartiles of all 3 androgen measures were observed for race, current menstrual status, oligomenorrhea symptom, age at menarche, or physical activity.

**Table 1.** Mean/percentage distribution of selected baseline characteristics of premenopausal women from the CARDIA study (1987-1988) by androgen quartiles

Variable, unit^a^	Androgen quartiles				P^b^
	Q1	Q2	Q3	Q4	
	Total testosterone (total T)				
Quartile (Q) values (ng/dL)	5-<27	27-<39	39-<53	53-150	
N	180	171	203	187	
Age (y)	26.2	26.3	25.7	25.8	.309
Race (% Black)	55.6	49.7	47.7	49.7	.471
Education (y)	14.4	14.4	14.4	14.0	.247
Current smoker (%)	11.7	17.0	24.1	36.9	<.001
Currently menstruating (%)	16.7	14.6	18.2	11.8	.328
Current OC/hormone user*^c^* (%)	62.2	35.7	24.1	18.2	<.001
BMI (kg/m^2^)	24.0	25.2	25.3	26.3	.003
Physical activity score	292.3	298.2	324.4	313.4	.544
Insulin (µU/ML)	10.8	11.7	11.7	12.4	.378
Oligomenorrhea status (%)	8.9	11.1	12.3	16.0	.201
Age at menarche	12.6	12.4	12.5	12.8	.073
PCOS (%)	3.9	3.5	4.9	10.7	.010
Insulin resistance*^d^* (%)	30.0	30.4	31.0	30.5	.997

	Free testosterone (free T)				
Quartile values (ng/dL)	0.01-<0.13	0.13-<0.25	0.25-<0.38	0.38-1.35	
N	166	199	180	196	
Age (y)	25.9	26.3	25.8	25.9	.518
Race (% Black)	53.0	45.2	53.9	51.0	.324
Education (y)	14.4	14.7	14.2	14.0	.007
Current smoker (%)	12.1	17.6	24.4	35.2	<.001
Currently menstruating (%)	13.9	20.6	14.4	12.2	.108
Current OC/hormone user*^c^* (%)	73.5	43.2	16.1	9.7	<.001
BMI (kg/m^2^)	23.5	24.5	25.1	27.5	<.001
Physical activity score	307.6	301.3	309.7	312.7	.971
Insulin (µU/ML)	10.4	11.0	11.1	14.0	<.001
Oligomenorrhea status (%)	10.2	10.1	11.7	16.3	.201
Age at menarche (y)	12.7	12.4	12.7	12.6	.361
PCOS (%)	4.2	2.5	5.0	11.2	.002
Insulin resistance*^d^* (%)	26.5	29.2	28.3	37.2	.110

	SHBG				
Quartile values (nmol/L)	11-<22	22-<30	30-<42	42-81	
N	182	185	183	191	
Age (y)	25.6	26.5	26.1	25.5	.021
Race (% Black)	57.7	47.6	50.3	47.1	.153
Education (y)	13.9	14.3	14.5	14.5	.012
Current smoker (%)	28.0	26.5	19.7	16.8	.026
Currently menstruating (%)	13.7	16.2	14.2	17.3	.753
Current OC/hormone user*^c^* (%)	11.5	15.1	31.2	78.5	<.001
BMI (kg/m^2^)	28.0	25.1	24.3	23.5	<.001
Physical activity score	278.2	327.5	310.9	313.9	.236
Insulin (µU/mL)	14.2	11.5	11.3	9.9	<.001
Oligomenorrhea status (%)	12.6	13.5	13.1	9.4	.604
Age at menarche	12.4	12.6	12.8	12.6	.266
PCOS (%)	9.3	6.0	4.9	3.1	.075
Insulin resistance*^d^* (%)	39.6	28.1	30.1	24.6	.014

Abbreviations: BMI, body mass index; HOMA, homeostatic model assessment; IR, insulin resistance; OC, oral contraceptive; PCOS, polycystic ovary syndrome.

^*a*^Numbers are means unless otherwise noted.

^*b*^ *P* values for overall group comparison based on F or χ^2^ tests.

^*c*^99.5% were OC users.

^*d*^Insulin resistance: HOMA-IR > 2.5, with HOMA-IR was calculated as glucose (mmol/L) × insulin (mU/L)/22.5.

In 23 years of follow-up, 224 women (30.2% of the study cohort) developed MetS. Table 2 shows adjusted hazard ratios (HR) for incident MetS during 23 years of follow-up across quartiles of each androgen measure. With adjustment for age, race, and education, the HRs (95% CIs) of developing MetS were 1.46 (1.02-2.10) and 2.22 (1.53-3.21) for women in the highest vs lowest total T and free T quartiles, respectively. The hazards of developing MetS were 47%, 59%, and 53% lower for women with SHBG in the second, third, and fourth quartiles (vs lowest quartile), respectively. Associations were attenuated for total T but remained for free T and SHBG, with further adjustments for smoking, physical activity, menstrual status, OCHM use, insulin level, oligomenorrhea symptom, and age at menarche. Furthermore, adding BMI into the models only partially attenuated the associations of free T and SHBG with MetS, but the associations still persisted (model III). We also observed similar associations in the models without individuals who reported using diabetes medication in any of the 8 examinations (results not shown).

**Table 2.** Adjusted*^a^* hazard ratios (95% CI) of cumulative incident metabolic syndrome (MetS) over 23 years of follow-up in premenopausal women from the CARDIA study by baseline (1987-1988) serum androgen quartiles

	Androgen quartiles				*P*-trend*^b^*
	Q1	Q2	Q3	Q4	
**Model I**					
Total testosterone	1.00	1.05 (0.70-1.56)	1.15 (0.79-1.68)	1.46 (1.02-2.10)	.035
Free testosterone	1.00	1.12 (0.74-1.69)	0.99 (0.65-1.52)	2.22 (1.53-3.21)	<.0001
SHBG	1.00	0.53 (0.38-0.75)	0.41 (0.29-0.60)	0.47 (0.32-0.67)	<.0001
**Model II**					
Total testosterone	1.00	0.96 (0.64-1.45)	1.08 (0.72-1.61)	1.30 (0.87-1.95)	.147
Free testosterone	1.00	1.04 (0.68-1.60)	1.04 (0.66-1.66)	2.10 (1.35-3.27)	.0003
SHBG	1.00	0.54 (0.38-0.77)	0.43 (0.29-0.63)	0.48 (0.31-0.73)	<.0001
**Model III**					
Total testosterone	1.00	0.93 (0.62-1.39)	0.96 (0.64-1.43)	1.03 (0.69-1.55)	.814
Free testosterone	1.00	0.98 (0.64-1.50)	0.92 (0.57-1.46)	1.58 (1.002-2.48)	.030
SHBG	1.00	0.65 (0.45-0.92)	0.52 (0.35-0.76)	0.60 (0.39-0.92)	.003

Abbreviation: CARDIA, Coronary Artery Risk Development in Young Adults.

^*a*^Model I was adjusted for year 2 age, race, and education; model II was adjusted for all variables in model I plus smoking, menstrual status, oral contraceptive/hormone use, physical activity score, insulin level, oligomenorrhea symptom, and age at menarche; model III was adjusted for all variables in model II plus body mass index.

^*b*^ *P* values for linear trends across serum androgen quartiles in Cox proportional hazard models.

Results from analyses conducted separately for Blacks and Whites showed no difference by race in patterns of associations of all 3 androgen measures with development of MetS (Table 3); the interaction terms for race with each androgen measure were also not statistically significant in the overall model (*P*-interaction terms >.05). In analyses stratified by OCHM use (Table 4), the associations for free T and SHBG were similar in magnitude to the main analysis among OCHM nonusers, but no associations were observed for OCHM users (*P*-interaction terms = .004).

**Table 3.** Adjusted*^a^* hazard ratios (95% CI) of cumulative incident metabolic syndrome (MetS) over 23 years of follow-up in premenopausal women from the CARDIA study by baseline (1987-1988) serum androgen quartiles and by race

	Androgen quartiles				*P*-trend*^b^*
	Q1	Q2	Q3	Q4	
**Whites (n = 366)**					
Total testosterone	1.00	0.75 (0.36-1.57)	1.19 (0.60-2.38)	1.16 (0.55-2.45)	.414
Free testosterone	1.00	0.67 (0.33-1.40)	1.12 (0.51-2.42)	1.67 (0.78-3.58)	.061
SHBG	1.00	0.49 (0.27-0.91)	0.45 (0.23-0.89)	0.43 (0.21-0.89)	.017
**Blacks (n = 375)**					
Total testosterone	1.00	1.10 (0.67-1.80)	1.05 (0.64-1.74)	1.38 (0.85-2.23)	.223
Free testosterone	1.00	1.37 (0.80-2.34)	1.03 (0.57-1.85)	2.38 (1.38-4.12)	.002
SHBG	1.00	0.52 (0.33-0.80)	0.41 (0.26-0.66)	0.50 (0.29-0.87)	.001

Abbreviation: CARDIA, Coronary Artery Risk Development in Young Adults.

^*a*^Models were adjusted for baseline age, education, smoking, menstrual status, oral contraceptive/hormone use, physical activity score, insulin level and age at menarche.

^*b*^ *P* values for linear trends across serum androgen quartiles in Cox proportional hazard models.

**Table 4.** Adjusted*^a^* hazard ratios (95% CI) of cumulative incident metabolic syndrome (MetS) over 23 years of follow-up in premenopausal women from the CARDIA study by baseline (1987-1988) serum androgen quartiles and by oral contraceptive/exogenous hormone use

	Androgen quartiles				*P*-trend*^b^*
	Q1	Q2	Q3	Q4	
**OCHM users (n = 256)**					
Total testosterone	1.00	1.30 (0.69-2.43)	1.05 (0.50-2.23)	1.76 (0.90-3.44)	.162
Free testosterone	1.00	0.84 (0.47-1.49)	0.89 (0.41-1.96)	1.82 (0.86-3.82)	.315
SHBG	1.00	0.55 (0.20-1.53)	0.54 (0.23-1.31)	0.87 (0.40-1.88)	.660
**Non-OCHM users (n = 485)**					
Total testosterone	1.00	0.82 (0.47-1.41)	1.01 (0.61-1.68)	1.12 (0.67-1.87)	.387
Free testosterone	1.00	1.18 (0.57-2.42)	1.07 (0.53-2.16)	2.17 (1.11-4.25)	.001
SHBG	1.00	0.55 (0.38-0.80)	0.41 (0.26-0.64)	0.23 (0.09-0.58)	<.001

Abbreviations: CARDIA, Coronary Artery Risk Development in Young Adults; OCHM, oral contraceptive/hormone.

^*a*^Models were adjusted for baseline age, race, education, smoking, menstrual status, physical activity score, insulin level, and age at menarche.

^*b*^ *P* values for linear trends across serum androgen quartiles in Cox proportional hazard models.

The associations of total T, free T, and SHBG with individual MetS components are shown in Table 5. In general, with full adjustment for covariates, the relations of free T and SHBG with individual MetS components were similar to the relations of free T and SHBG with MetS. Both measures were significantly related to greater hazard of incident central adiposity and hypertension. Women with SHBG levels in the higher quartiles (vs the lowest) also had significantly lower hazards of developing low HDL cholesterol by 28 to 39%. There was also a linear trend for high triglycerides, with a higher quartile of SHBG associated with a lower hazard of high triglycerides (*P*-trend = .042). Total T, free T, and SHBG were not significantly related to the development of elevated glucose levels.

**Table 5.** Adjusted*^a^* hazard ratios (95% CI) of cumulative incident metabolic syndrome (MetS) components from over 23 years of follow-up in premenopausal women from the CARDIA study by baseline (1987-1988) serum androgens quartiles

MetS components*^b^*	Androgen quartiles				*P*-trend*^c^*
	Q1	Q2	Q3	Q4	
**Total testosterone**					
High triglycerides	1.00	0.88 (0.56-1.38)	0.84 (0.54-1.32)	0.98 (0.62-1.55)	.937
Low HDL	1.00	1.12 (0.84-1.49)	0.95 (0.71-1.27)	1.01 (0.75-1.37)	.782
Hypertension	1.00	1.07 (0.77-1.49)	1.21 (0.87-1.69)	1.00 (0.71-1.41)	.873
Abnormal glucose	1.00	1.61 (1.03-2.52)	1.39 (0.88-2.19)	1.67 (1.06-2.63)	.073
Abdominal obesity	1.00	1.09 (0.81-1.46)	1.25 (0.93-1.67)	1.25 (0.92-1.70)	.107
**Free testosterone**					
High triglycerides	1.00	0.61 (0.38-0.97)	0.77 (0.47-1.25)	1.03 (0.64-1.68)	.444
Low HDL	1.00	1.02 (0.76-1.37)	1.02 (0.74-1.41)	1.35 (0.98-1.86)	.059
Hypertension	1.00	1.11 (0.79-1.57)	1.03 (0.70-1.51)	1.60 (1.10-2.33)	.015
Abnormal glucose	1.00	1.22 (0.77-1.94)	1.20 (0.73-1.97)	1.62 (0.99-2.66)	.057
Abdominal obesity	1.00	1.04 (0.77-1.43)	1.34 (0.96-1.86)	2.02 (1.45-2.82)	<.0001
**SHBG**					
High triglycerides	1.00	0.76 (0.51-1.13)	0.66 (0.43-1.02)	0.64 (0.39-1.03)	.042
Low HDL	1.00	0.72 (0.55-0.94)	0.68 (0.52-0.89)	0.62 (0.45-0.84)	.002
Hypertension	1.00	0.70 (0.51-0.95)	0.55 (0.40-0.76)	0.62 (0.43-0.90)	.002
Abnormal glucose	1.00	0.90 (0.62-1.31)	0.67 (0.44-1.01)	0.79 (0.49-1.28)	.134
Abdominal obesity	1.00	0.48 (0.37-0.62)	0.41 (0.31-0.54)	0.41 (0.30-0.56)	<.0001

Abbreviations: CARDIA, Coronary Artery Risk Development in Young Adults; HDL, high-density lipoprotein.

^*a*^Adjusted for year 2 age, race, education, smoking, menstrual status, oral contraceptive/hormone use, physical activity score, insulin level, oligomenorrhea symptom, and age at menarche.

^*b*^High triglycerides: triglycerides ≥150 mg/dL; low HDL: HDL cholesterol < 50 mg/dL; hypertension: blood pressure ≥130/85 mm Hg or on treatment; abnormal glucose: fasting glucose ≥ 110 mg/dL or on treatment; abdominal obesity: waist circumference ≥ 88 cm.

^*c*^ *P* values for linear trends across serum androgen quartiles in Cox proportional hazard models.

In sensitivity analyses when education, smoking, physical activity, and OCHM use were modeled as time-varying covariates, results were generally similar to those based on fixed baseline covariates for free T and SHBG (Table 6). Moreover, we observed a significant linear trend across quartiles of total T (*P* = .032) and the hazards of developing MetS were significantly higher for those with the total T in the fourth quartile vs those in the first quartile (model I). The addition of weight change as a time-varying covariate into the models had no impact on the associations of androgens/SHBG and MetS (model II). When BMI was added as a time-varying covariate, the associations of free T and SHBG with MetS were slightly attenuated; nevertheless, these associations still remained (model III).

**Table 6.** Adjusted*^a^* hazard ratios (95% CI) of cumulative incident metabolic syndrome (MetS) over 23 years of follow-up in premenopausal women from the CARDIA study by baseline (1987-88) serum androgen quartiles with time-dependent covariates

	Androgen quartiles				*P*-trend*^b^*
	Q1	Q2	Q3	Q4	
**Model I**					
Total testosterone	1.00	1.15 (0.71-1.87)	1.36 (0.86-2.14)	1.60 (1.01-2.51)	.032
Free testosterone	1.00	1.44 (0.87-2.40)	1.12 (0.65-1.93)	2.55 (1.59-4.08)	<.0001
SHBG	1.00	0.63 (0.42-0.95)	0.38 (0.24-0.61)	0.47 (0.30-0.73)	<.0001
**Model II**					
Total testosterone	1.00	1.16 (0.72-1.89)	1.38 (0.88-2.18)	1.60 (1.02-2.52)	.031
Free testosterone	1.00	1.46 (0.88-2.43)	1.12 (0.65-1.92)	2.58 (1.61-4.13)	.0001
SHBG	1.00	0.62 (0.41-0.93)	0. 38 (0.24-0.60)	0.47 (0.30-0.73)	<.0001
**Model III**					
Total testosterone	1.00	1.20 (0.74-1.94)	1.41 (0.90-2.22)	1.35 (0.85-2.14)	.154
Free testosterone	1.00	1.39 (0.83-2.30)	1.14 (0.66-1.95)	2.02 (1.25-3.27)	.006
SHBG	1.00	0.73 (0.48-1.10)	0.49 (0.31-0.78)	0.61 (0.39-0.96)	.005

Abbreviation: CARDIA, Coronary Artery Risk Development in Young Adults.

^*a*^Model I was adjusted for age, race, menstrual status, insulin level at baseline, oligomenorrhea symptom, and age at menarche; and time-varying education, smoking, oral contraceptive/hormone use status, and physical activity score; model II was adjusted for all variables in model I and weight change; model III was adjusted for all variables in model I and body mass index.

^*b*^ *P* values for linear trends across serum androgen quartiles in Cox proportional hazard models.

In analyses using androgens/SHBG measured at examination years 10 and 16, respectively, patterns of association of free T and SHBG, measured at year 10 (177 incident MetS cases; 23% of the sample) and year 16 (99 incident MetS cases; 18% of the sample), with incident MetS were similar to those with androgens measured from baseline, despite the shorter follow-up and fewer cases of incident MetS. For example, HRs (95% CIs) of developing MetS were 2.41 (1.51-3.84) and 2.15 (1.20-3.86) for androgens/SHBG measured in year 10 and year 16, respectively, for those with free T in the fourth quartile vs those in the first quartile (Table 7).

**Table 7.** Adjusted*^a^* hazard ratios (95% CI) of cumulative incident metabolic syndrome (MetS) over 15 and 9 years of follow-up in premenopausal women from the CARDIA study by year 10 (1995-96) and year 16 (2002-03) serum androgen quartiles

	Androgen quartiles				*P*-trend*^b^*
	Q1	Q2	Q3	Q4	
**Year 10**					
Total testosterone	1.00	0.87 (0.55-1.38)	0.84 (0.53-1.32)	1.44 (0.95-2.17)	.080
Free testosterone	1.00	1.30 (0.78-2.18)	1.58 (0.95-2.63)	2.41 (1.51-3.84)	<.0001
SHBG	1.00	0.67 (0.47-0.96)	0.40 (0.26-0.61)	0.25 (0.15-0.42)	<.0001
**Year 16**					
Total testosterone	1.00	1.07 (0.59-1.93)	0.98 (0.52-1.85)	1.32 (0.74-2.36)	.366
Free testosterone	1.00	0.89 (0.46-1.70)	0.90 (0.45-1.78)	2.15 (1.20-3.86)	.003
SHBG	1.00	0.42 (0.26-0.70)	0.39 (0.23-0.66)	0.15 (0.07-0.32)	<.0001

Abbreviation: CARDIA, Coronary Artery Risk Development in Young Adults.

^*a*^Models were adjusted for baseline age, race, education, smoking, menstrual status, physical activity score, insulin level, oral contraceptive/hormone use status, and age at menarche.

^*b*^ *P* values for linear trends across serum androgen quartiles in Cox proportional hazard models.

Finally, analyses using year 2 and year 16 data for those without PCOS (n = 698 for year 2 and n = 537 for year 16) produced similar associations of androgenicity and MetS (Table 8) to associations shown in all women (Table 2 for year 2 and Table 7 for year 16).

**Table 8.** Adjusted*^a^* hazard ratios (95% CI) of cumulative incident metabolic syndrome (MetS) over 23 and 9 years of follow-up in premenopausal without PCOS women from the CARDIA study by year 2 (1987-88) and year 16 (2002-03) serum androgen quartiles

	Androgen quartiles				*P*-trend*^b^*
	Q1	Q2	Q3	Q4	
**Year 2**					
Total testosterone	1.00	1.01 (0.67-1.53)	1.11 (0.73-1.67)	1.36 (0.90-2.05)	.124
Free testosterone	1.00	1.08 (0.70-1.67)	1.09 (0.68-1.75)	2.23 (1.42-3.51)	.002
SHBG	1.00	0.55 (0.39-0.79)	0.41 (0.28-0.62)	0.49 (0.31-0.75)	<.0001
**Year 16**					
Total testosterone	1.00	0.98 (0.53-1.80)	1.04 (0.54-1.99)	1.29 (0.71-2.32)	.343
Free testosterone	1.00	0.88 (0.45-1.71)	0.86 (0.42-1.73)	2.17 (1.20-3.95)	.003
SHBG	1.00	0.43 (0.25-0.71)	0.39 (0.23-0.68)	0.16 (0.07-0.34)	<.0001

Abbreviation: CARDIA, Coronary Artery Risk Development in Young Adults.

^*a*^Models were adjusted for baseline age, race, education, smoking, menstrual status, physical activity score, insulin level, oral contraceptive/hormone use status, and age at menarche.

^*b*^ *P* values for linear trends across serum androgen quartiles in Cox proportional hazard models.

## Discussion

Using data drawn from female participants in a large population-based biracial cohort, we found that after 23 years of follow-up, higher free T levels and lower SHBG levels measured in young adulthood were associated with higher risk of developing MetS before menopause, independent of known risk factors for MetS. Patterns of these associations were similar for both Blacks and Whites, and for OCHM nonusers, but not for OCHM users.

To our knowledge, this is the first population-based, longitudinal, observational study reporting decades-long associations of androgen levels with incident MetS before menopause in young Black and White women. Our findings were in line with the findings from previous longitudinal studies that focused on these associations with shorter follow-up time and/or in older premenopausal women. The Study of Women’s Health Across the Nation with 1862 midlife multiracial women (mean age 46 years) also revealed that higher baseline total T and free androgen index (FAI) (calculated based on the measured total T and SHBG levels), as well as lower SHBG levels were associated with higher incident MetS after 5 years of follow-up. Multivariable-adjusted relative risks (95% CIs) of incident MetS for total T, FAI, and SHBG were 2.04 (1.15-2.86), 1.77 (1.50-2.09), and 0.58 (0.48-0.70), respectively. The Study of Women’s Health Across the Nation also did not find any differences in the associations by race/ethnicity [20].

Another longitudinal study with a short follow-up time and only on White women—the Study of Health in Pomerania in northeastern Germany—analyzed data from 1111 pre- and 963 postmenopausal German women and found that free T was positively, and SHBG was inversely, associated with 5-year incident MetS in their age-adjusted models only—relative risks (95% CIs) were 1.32 (1.11-1.56) and 0.61 (0.51-0.73), respectively. In multivariable-adjusted models stratifying by menopausal status, they found no significant associations of SHBG or other endogenous androgens with 5-year incidence of MetS among premenopausal women, although they found SHBG was inversely associated with incident MetS among postmenopausal women. However, it should be noted that the study’s results stratifying by menopausal status came from models adjusted for waist circumference (where the high level of waist circumference is 1 of the MetS components) [25], hence the results may not reflect the true association of androgen levels and incident MetS.

A few previous studies examining long-term associations of androgens and SHBG with individual components of MetS or cardiometabolic diseases have also yielded similar findings to ours. For instance, a recent prospective study examining associations of hyperandrogenemia in early adulthood and the risk of abnormal glucose metabolism after 15 years of follow-up in 3280 middle-aged women in Northern Finland found that highest T and FAI quartiles and lower SHBG quartiles were related to higher odds of abnormal glucose metabolism (ie, prediabetes and type 2 diabetes mellitus) [18]. Another prospective study of 1462 Swedish women baseline aged 38 to 60 years with 12 years of follow-up found that low SHBG level was associated with a higher age-adjusted risk of developing diabetes mellitus [24]. In our study, we did not observe any association between SHBG and elevated glucose level in younger premenopausal women.

Although some studies report that the prevalence of MetS may differ by ethnicity [36, 37], we found that patterns of associations of androgens and SHBG levels with incident MetS were similar for both Black and White women. However, we observed that the associations of free T and SHBG with incident MetS were only present among OCHM nonusers but not among OCHM users (most were OC users in our study). This may be due to the effects of OC use on the levels of androgens and SHBG, as previously reported [35]. Consequently, the association of androgens and SHBG with MetS can be obscured among OC users.

It remains unclear whether higher androgen levels are a causal factor in the development of MetS or merely serve as a marker for primary hormonal or metabolic disturbances leading to MetS. Much of the evidence for the atherogenicity of androgens in women has been derived from studies on women with PCOS that have consistently demonstrated a clustering of CVD risk factors independent of obesity in PCOS patients. Obesity-related changes in insulin and bioavailable IGF-1 have been shown to stimulate ovarian androgen synthesis. On the other hand, androgen receptors have been identified on adipocytes with evidence that androgens may influence intra-abdominal fat development [38], and lowering of androgen levels in hyperandrogenic women has been shown to improve insulin sensitivity and the lipid profile [39-42]. We previously reported in our CWS study population that higher BMI and larger waist circumference were inversely associated, cross-sectionally, with SHBG and directly associated with free T [42]. Furthermore, changes in BMI and SHBG over a 14-year follow-up period may occur concurrently rather than sequentially [42]. However, in this current study, the associations of androgenicity with development of MetS were independent from insulin levels, BMI level, diabetes medication use, and weight change.

Our findings benefited from being able to examine decades-long associations of androgenicity with MetS and its components in a sample of healthy young biracial premenopausal women. It is also important to note that although the relationship of MetS to androgen excess disorders, primarily PCOS, has been well documented, our findings demonstrate a graded direct association of testosterone and an inverse association of SHBG levels with risk of MetS exist starting well within the clinically accepted normal range.

Limitations of this study include only having direct measurements of total T and SHBG, hence free T was derived and other androgens could not be examined. However, these measurements used state-of-the-art measurements available during the study period (early 2000s). Total T was measured by immunoassay rather than the liquid chromatography-tandem mass spectrometry assays, and this could potentially impact the robustness of the findings [43]. Even with these limitations acknowledged, we and others have consistently observed numerous associations of these measures with cardiometabolic phenotypes [42, 44, 45].

In conclusion, our findings suggest that among healthy premenopausal young women, elevated androgenicity is associated with development of MetS. These findings, together with the results from our previous study on the associations of androgens and BMI [42] and subclinical CVD [44] once more demonstrate the potential role of androgens in relationship to women’s health outcomes. Early assessment of androgen levels may provide an opportunity to identify women at higher risk for MetS who can benefit from rigorous lifestyle modifications, particularly weight loss, regular physical activity, and a healthy diet, with the potential to prevent MetS, diabetes, and CVDs later in life.

## Data Availability

Some or all datasets generated during and/or analyzed during the current study are not publicly available but are available from the corresponding author on reasonable request.

## Abbreviations

BMI: body mass index
CARDIA: Coronary Artery Risk Development in Young Adults
CVD: cardiovascular disease
CWS: CARDIA Women’s Study
FAI: free androgen index
HDL: high-density lipoprotein
HR: hazard ratio
MetS: metabolic syndrome
OCHM: oral contraceptive/exogenous hormone
PCOS: polycystic ovarian syndrome
T: testosterone
